# From Intermediate Epoxy Group to Stable Ether Bridge: Insights from DFT Study on Graphene Quantum Dots

**DOI:** 10.3390/molecules31132269

**Published:** 2026-06-29

**Authors:** Dmitry Romanov, Anatoly Lavrentyev, Igor Ershov

**Affiliations:** 1 Department of Electrical Engineering and Electronics, Don State Technical University, Rostov-on-Don 344000, Russia; 2 Department of Physics, Don State Technical University, Rostov-on-Don 344000, Russia; iershov@donstu.ru

**Keywords:** graphene quantum dots, Clar’s rule, epoxy group, ether bridge, DFT, transition states

## Abstract

This study investigates the mechanism of ether bridge formation on the edges of graphene quantum dots (GQDs) and evaluates its impact on their structural, electronic, and optical properties. Using density functional theory (DFT) coupled with Clar’s aromatic sextet rule, we analyzed different edge functionalization sites on a model nanographene. The kinetic parameters evaluated via the Eyring–Polanyi equation demonstrate that the stability of functional groups is fundamentally governed by the retention or migration of aromatic sextets. While epoxidation at thermodynamically favorable edge sites that form stable epoxy intermediates exhibits high kinetic stability with substantial activation barriers, alternative configurations directly relax during geometry optimization to minimize aromaticity disruption. Moreover, highly metastable epoxy intermediates convert to ether bridges via nearly barrierless pathways at ambient temperature. Simplified time-dependent DFT (sTD-DFT) calculations show that oxygen functionalization narrows the energy gap, yielding a distinct bathochromic shift into the visible range. Ultimately, Clar’s rule is established as a predictive tool for ether bridge formation, enabling the rational design of GQDs with tailored stability and optical properties for bioimaging and optoelectronic applications.

## 1. Introduction

Graphene quantum dots (GQDs) are promising materials for optoelectronics [1], sensing [2], and bioimaging [3] due to their unique electronic and optical properties [4]. However, a prerequisite for the practical application of GQDs is the ability to precisely tune their physicochemical characteristics.

One of the promising methods for tuning the final properties of GQDs is their modification with oxygen-containing groups [5,6]. In particular, the epoxidation of GQD edge sites serves as an effective tool for adjusting their optical characteristics. Specifically, epoxy group attachment can induce changes in the energy gap and lead to shifts in absorption and emission bands [7]. Furthermore, GQD epoxidation can enhance spin–orbit coupling [8] and reduce the energy difference between singlet and triplet states, thereby increasing the probability of intersystem crossings and facilitating the efficient population of triplet levels. Thus, epoxidation, by enabling control over the spectral characteristics of GQDs, improves their efficacy in bioimaging applications as well as photodynamic and photothermal therapies [9].

It is important to note that the electronic and optical properties of GQDs are largely determined by their delocalized π-electron system. Consequently, the effectiveness of tuning GQD optical characteristics depends on the epoxidation site: epoxy functionalization induces a change in carbon atom hybridization from sp^2^ to sp^3^, disruption of π-conjugation, and loss of GQD planarity [10].

Furthermore, epoxidation induces significant local strain: the bond angle in the three-membered epoxide ring is approximately 60°, which critically deviates from the ideal tetrahedral angle (~110°). Consequently, the epoxy group exhibits relatively low stability and high reactivity upon contact with air or solvent [11]. As a result, epoxy groups demonstrate a strong tendency to transform into other functional groups, characterizing them as an intermediate form of functionalization whose stability depends on external conditions [12]. Thus, the uncontrolled transformation of the epoxy group prevents achieving the intended effects of epoxidation, thereby reducing its efficacy for tuning GQD characteristics.

However, it is notable that an alternative transformation pathway independent of the external environment also exists: C–C bond cleavage between epoxidized carbon atoms [13]. As a result, the epoxy group transforms into an ether bridge characterized by lower reactivity: carbon atom hybridization reverts to sp^2^, partially restoring π-electron delocalization and reducing local strain in GQDs [14]. Currently, the mechanism of ether bridge formation and its influence on the electronic and optical properties of structures remain insufficiently studied.

Consequently, the objectives of this work are to determine the GQD positions most susceptible to ether bridge formation, identify the origins of the transformation, and assess its contribution to the structural, electronic, and optical characteristics of nanostructures. Since the stability of GQDs is directly related to the preservation of their aromaticity, Clar’s aromatic sextet rule [15,16,17] was chosen as the key analysis tool. The applicability of the selected approach was demonstrated in our recent study [14]: it was shown that Clar’s rule is an effective predictive tool for evaluating the stability and electronic characteristics of epoxidized GQDs, despite the disruption of their planarity.

## 2. Results and Discussion

**Figure 1:**
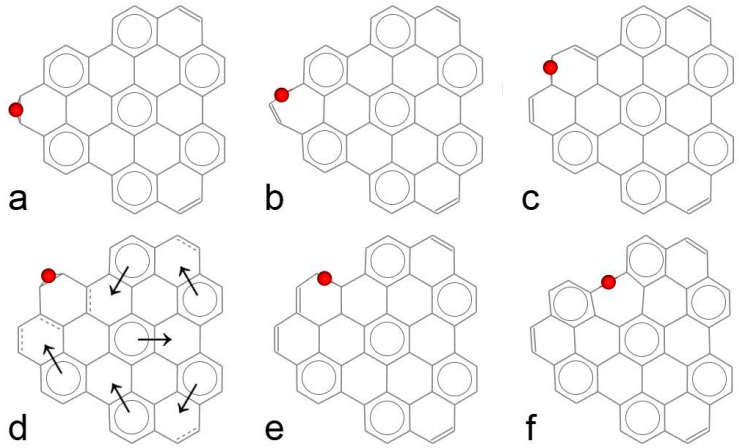
Oxygen attachment sites on GQD-D3h. (**a**) α position (epoxy-α), (**b**) β position (ether-β), (**c**) γ position (epoxy-γ), (**d**) δ position (epoxy-δ), (**e**) ε position (epoxy-ε), (**f**) ζ position (ether-ζ). Aromatic sextet positions are denoted by circles; their migration pathways are indicated by arrows.

Given the high reactivity of GQD edge sites [18], an important criterion for selecting a model structure is the presence of a large variety of nonequivalent positions for functionalization. For this reason, a nanographene with the chemical composition C_48_H_18_ and symmetry point group D_3h_ (GQD-D3h), featuring mixed edge types (zigzag and armchair) [19,20], was employed as the model structure of pristine graphene quantum dots. The D_3h_ symmetry point group enables the identification of six unique edge positions for epoxy group attachment to the GQD surface. The specific oxygen configurations on GQD-D3h after geometry optimization are depicted in Figure 1. Aromatic sextet positions are denoted by circles, with arrows indicating their migration pathways. The general mechanism of an epoxy group transformation into an ether bridge is presented in Figure S1 (in Supplementary Materials). The calculated absolute and relative oxygen binding energy, energy gap, symmetry group and transformation barrier of epoxidized GQD D3h forms are summarized in Table 1. The total binding energies of the structures are presented in Table S1 (in Supplementary Materials).

Regardless of the attachment position, oxygen adsorption induces energy gap narrowing in GQD-D3h (the pristine structure exhibits a gap of 3.19 eV). This gap reduction causes a bathochromic shift in the absorption bands into the visible spectral range upon functionalization. The optical absorption spectra of epoxidized structures are presented in Figure S2 of the Supplementary Materials.

As shown in Table 1, the energy barrier for the transformation of the epoxy group into an ether bridge depends significantly on the functionalization position. Thus, GQD-D3h epoxidized at positions α and δ exhibits low susceptibility to transformation and high kinetic stability: these positions are characterized by high oxygen binding energy and energy barrier values. Overcoming these barriers requires substantial thermal activation. Our estimates using transition state theory (TST) and the values of Gibbs free energy of activation at 298 K (Table 1) give the temperatures for the transformation into an ether bridge on the order of 570–620 K for position epoxy-α and approximately 620–670 K for the position epoxy-δ. Conversely, the extremely low barriers for GQD-D3h epoxidized at positions epoxy-γ and epoxy-ε indicate metastability and the tendency to transform at any finite temperatures. Positions epoxy-β and epoxy-ζ are equally notable: geometry optimization via singlet oxygen atom adsorption leads directly to the formation of ether bridges, indicating the absence of a local minimum corresponding to the epoxy configuration on the potential energy surface.

The α epoxidation position is the most energetically favorable and exhibits low susceptibility to transformation into an ether bridge. High oxygen binding energy and a significant energy barrier correlate with the preservation of the aromaticity of the system. As shown in Figure 1, the oxygen atom in the epoxy-α position does not disrupt the aromatic sextets, thereby preserving their number and uniform arrangement.

The epoxidation of GQD-D3h at the δ position exhibits negligible changes in the oxygen binding energy, energy gap, and energy barrier of the structure compared to the α position. Despite the loss of one aromatic sextet, the epoxy group at the δ position does not cause significant disruption of the delocalized π-electron system: the loss of GQD-D3h local aromaticity is compensated by the enabled migration of the remaining aromatic sextets. Thus, it can be suggested that the migration of aromatic sextets, as well as their number, exerts a significant influence not only on structure energy and the energy gap but also on the transformation barrier value.

**Figure 2:**
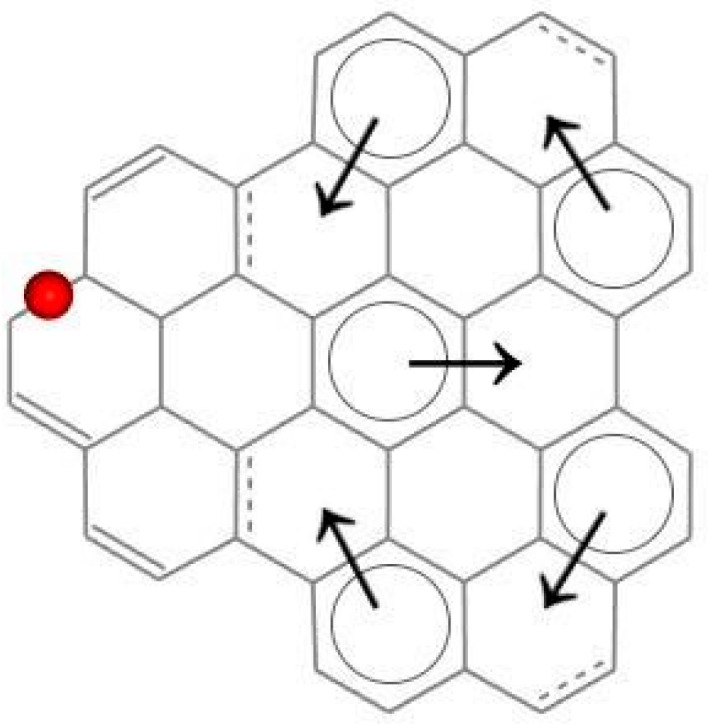
Position epoxy-β without the transformation of the epoxy group into an ether bridge. Aromatic sextet positions are denoted by circles; their migration pathways are indicated by arrows.

Unlike positions α and δ, epoxy group formation at positions β and ζ affects not one but two benzene rings. For this reason, the epoxy group at these positions is hypothetically expected to destroy two aromatic sextets. For position β, this is less evident: unlike position ζ, the oxygen atom affects only one aromatic sextet. However, epoxy group attachment induces π-electron localization in neighboring benzene rings, consequently resulting in the loss of another aromatic sextet. Figure 2 shows position β without the transformation of the epoxy group into an ether bridge. Aromatic sextet positions are denoted by circles; their migration pathways are indicated by arrows.

Since the loss of a large number of sextets adversely affects GQD stability, the system tends to minimize aromaticity disruption. Forming an ether bridge at positions β and ζ maintains the sp^2^ hybridization of the carbon atoms. This effectively prevents π-electron localization in adjacent rings, thereby maximizing the number of preserved aromatic sextets.

It is also notable that ether bridge formation in these positions occurs through the cleavage of a formally single σ-bond. Specifically, at position epoxy-ζ, the C–C bond connecting two aromatic sextets breaks, preventing it from being a localized π-bond. Unlike position ζ, the C–C bond at position β connects one aromatic sextet to a carbon atom already participating in localized π-bond formation in an adjacent benzene ring. Consequently, the bond susceptible to cleavage at position β is also formally single. As a result, bond cleavage between the functionalized carbon atoms represents an energetically more favorable and preferred relaxation pathway than the formation of an epoxy group (which can be observed in the geometry convergence plot, presented in Figure S3b,f in Supplementary Materials).

Consequently, ether bridge formation at positions β and ζ exerts a comparable influence on GQD-D3h aromaticity relative to epoxy formation at positions α and δ. However, concurrent C–C bond cleavage reduces the total number of covalent bonds and increases overall structural curvature, moderately decreasing the oxygen binding energy in ether-bridged structures. Simultaneously, the curvature of benzene rings occurs relatively uniformly (particularly at position ether-ζ due to Cs symmetry), preserving the bond angles necessary for π-electron delocalization. Thus, the bond angles of carbon atoms comprising the aromatic sextet affected by functionalization at position ether-β vary in the range of 117–122°, and for position ether-ζ, the range is 116–124°, which is formally sufficient for effective p-orbital overlap and resonance energy formation.

Additionally, the ether bridge at positions β and ζ exerts a comparable influence on the energy gap of the structure to that of the α epoxidation position: these positions exhibit the largest energy gaps compared to other epoxidized forms of GQD-D3h. The preservation of aromatic sextets reduces the influence of the oxygen atom on the frontier molecular orbitals of the structure, thereby preventing their significant redistribution. Thus, the formation of an ether bridge enables not only the preservation of relatively high structural stability but also the retention of an energy gap close to that of the pristine structure. The contributions of the epoxy group and the ether bridge to the HOMO and LUMO formation, analyzed via density of states (DOS), are depicted in Figure S4 of the Supplementary Materials. The spatial distribution of the frontier molecular orbitals for the pristine GQD-D3h and its functionalized forms is presented in Figure S5 of the Supplementary Materials.

To confirm the generalizability of the conclusions drawn for positions β and ζ to nanographenes of different sizes and symmetries, geometry optimization was additionally performed on benzo[a]pyrene—a molecule with the chemical composition C_20_H_12_ and symmetry point group C_1_. The epoxy group attachment sites on benzo[a]pyrene that yield ether bridges upon relaxation, under conditions analogous to positions β and ζ, are depicted in Figure S6 of the Supplementary Materials.

The results obtained for benzo[a]pyrene confirmed the hypothesis that aromatic sextets disruption induces ether bridge formation during structural relaxation. Specifically, epoxidized benzo[a]pyrene also undergoes C–C bond cleavage to avoid the loss of aromaticity: epoxy group formation causes destruction, while the ether bridge preserves aromatic sextets.

The epoxidation of GQD-D3h at positions γ and ε leads to a significant decrease in oxygen binding energy and the energy gap compared to the most stable epoxy-α configuration. In this case, the epoxy group destroys only one aromatic sextet, similarly to position δ. However, unlike δ, epoxidation at positions γ and ε results in π-electron localization in adjacent benzene rings, preventing sextet migration. Consequently, a significant loss of GQD-D3h aromaticity occurs, rendering the structure unstable.

**Figure 3:**
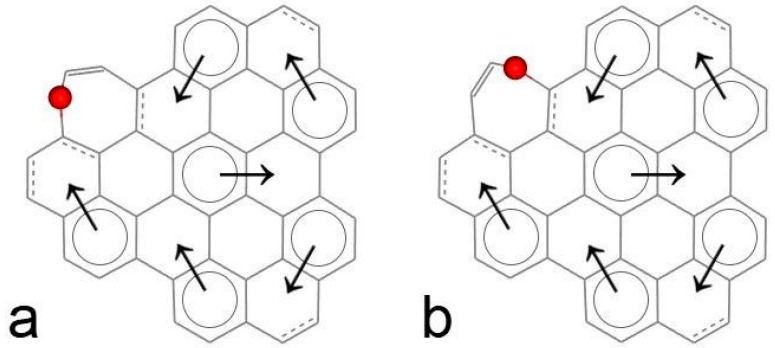
Positions ether-γ and ether-ε upon the transformation of the epoxy group into an ether bridge in GQD-D3h. (**a**) The ether-γ position, (**b**) the ether-ε position. Aromatic sextet positions are denoted by circles; their migration pathways are indicated by arrows.

In this case, the transformation of the epoxy group into an ether bridge at positions γ and ε does not prevent aromatic sextet destruction. Nevertheless, C–C bond cleavage restores π-electron delocalization in the adjacent ring, restoring the sextet migration capability. Thus, despite sextet destruction, aromaticity restoration compensates for the energy cost of bond cleavage, resulting in a low activation barrier. Positions γ and ε upon the transformation of the epoxy group into an ether bridge are depicted in Figure 3. Aromatic sextet positions are denoted by circles; their migration pathways are indicated by arrows. The oxygen binding energy, energy gap, and symmetry group for positions ether-γ and ether-ε upon transformation are summarized in Table 2.

For the epoxy-γ to ether-γ rearrangement, the calculated Gibbs free energy of activation at 298 K is only +0.2 kcal/mol (Table 1). The corresponding first-order rate constant (4.7 × 10^12^ s^−1^) yields a half-life of ~0.15 ps, indicating a reaction that is limited only by the vibrational timescale of the molecule. Thus, this transformation is effectively barrierless at all experimentally relevant temperatures; the ether-bridged product will form spontaneously as soon as the epoxy intermediate is generated, without requiring heating. Moreover, at 298 K, the Gibbs free energy of activation becomes negative for the epoxy-ε to ether-ε transformation, due to a substantial entropy gain in the loose transition state. While a negative barrier is unphysical in the strict TST sense, it unequivocally indicates that the process is barrierless for all practical purposes and will occur at a rate limited only by molecular vibrations. Consequently, the ether-ε product is expected to form spontaneously at ambient temperature and does not require thermal activation.

Following transformation, the arrangement of aromatic sextets, their migration pathways, and the energy gap for structures with an oxygen atom at positions ether-γ and ether-ε coincide with those at position epoxy-δ. However, C–C bond cleavage reduces the oxygen binding energy, rendering structures with an ether bridge marginally less stable compared to position δ (by ~3 kcal/mol) but similarly stable to those at positions β and ζ.

Based on our calculated oxygen binding energies, the epoxy adducts at the α and δ edge sites (epoxy-α and epoxy-δ positions) exhibit the most exothermic binding (−65.7 kcal/mol and −63.6 kcal/mol) among all surveyed positions. These positions also correspond to the highest edge reactivity predicted by previous theoretical studies on polycyclic aromatic hydrocarbons [7,10]. We therefore designate these isomers as the thermodynamically most plausible candidates for experimental realization in vacuum. However, during synthesis and processing in solution, epoxy groups exhibit lower kinetic stability compared to ether configurations; therefore, configurations ether-β and ether-ε are expected to be more favorable in solution.

Thus, an analysis of the energy gap for metastable (epoxy) configurations is inadequate for describing the actual properties of nanographenes. The significant gap variation characteristic of the epoxy group at these positions is not realized due to the spontaneous transformation into an ether bridge even at low temperatures. Transformed structures exhibit comparable energy gap values, resulting in the convergence of absorption band edge positions. The energy gap and optical properties (wavelength and oscillator strength for first two singlet excited states) of epoxidized GQD-D3h at various positions are summarized in Table 3.

Table 3 presents the energy gaps alongside the transition wavelengths, corresponding oscillator strengths and transition components for the two lowest singlet excitations, S_0_ → S_1_ and S_0_ → S_2_. Overall, we observe a spectral pattern typical of polycyclic aromatic hydrocarbons, characterized by two lowest-lying singlet excitations that give rise to the α and *p* absorption bands (according to Clar’s notation; see absorption spectra presented in Figure S2 in the Supplementary Materials). The α-band is usually very weak and has the lowest energy (S_0_ → S_1_ transition). The much more intense p-band originates from the HOMO → LUMO transition and is formed by the S_0_ → S_2_ excitation. However, the energetic ordering of these bands is inverted in epoxy-α, ether-β, and ether-ζ structures, as can be seen from the analysis of transition components (Table 3), which makes them potentially interesting as light emitters. Interestingly, the ordering of the S_1_ and S_2_ states is not inverted in the structures that enabled sextet migration.

While we are not aware of published UV-vis spectra for isolated epoxide or ether derivatives of C_48_H_18_, oxygen-functionalized graphene quantum dots of similar size have been characterized both theoretically and experimentally. The general trend observed in oxidized PAHs is as follows: the attachment of epoxy groups introduces low-energy states, which consistently red-shift the first absorption band [7,21]. For instance, it was experimentally shown [22] that epoxidation forms additional extrinsic states within the intrinsic bandgap inducing red-shifts in PL peaks.

## 3. Materials and Methods

**Figure 4:**
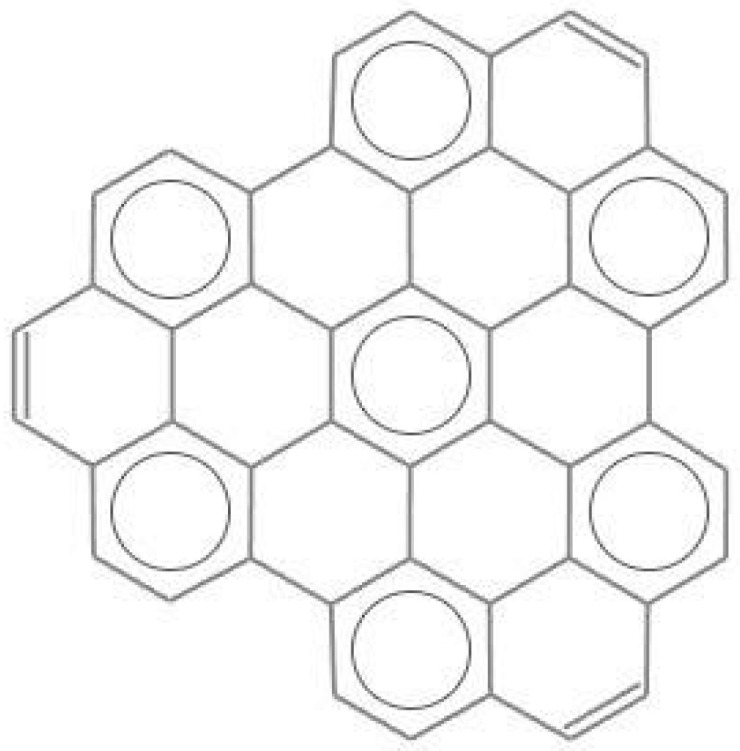
GQD-D3h with highlighted aromatic sextets. The positions of aromatic sextets are denoted by circles.

In the GQD-D3h structure, seven uniformly distributed aromatic sextets can be identified based on Clar’s rule, which indicates its high stability. GQD-D3h with highlighted aromatic sextets is shown in Figure 4, where the positions of aromatic sextets are denoted by circles.

To investigate the transformation of the epoxy group into an ether bridge on the surface of the GQD-D3h structure, quantum-chemical calculations were performed in the present study within the comprehensive framework of density functional theory (DFT) [23] using the all-electron Pople 6-311G(d,p) split-valence triple-zeta basis set with polarization functions on heavy atoms and hydrogen, which has previously demonstrated high accuracy in nanostructure calculations [24].

The full geometry optimization of pristine and epoxy-functionalized GQD-D3h structures was performed for a closed-shell singlet ground state using the hybrid B3LYP functional [25], which is widely employed in quantum-chemical calculations of GQDs. Transition state searches for the transformation of the epoxy group into an ether bridge were also carried out using the same functional within the nudged elastic band (NEB) method [26]. The analytic second derivatives of hybrid DFT energies were calculated to confirm the exact nature of stationary and metastable points [27]. The quantitative evaluation of activation temperatures was performed using transition state theory (TST) and the Eyring–Polanyi equation [28]. We used the half-life of the reactions in the range from 10 min to one hour for the evaluation of the reaction rates.

To adequately account for van der Waals dispersion interactions, the functional B3LYP was used with the atom-pairwise dispersion correction DFT-D3(BJ) [29]. All calculations were performed within the ORCA 6.1 program system [30]. An SCF convergence threshold of 1 × 10^−8^ Ha was used for all calculations. Geometry optimizations were performed with the following convergence criteria: energy change 5 × 10^−6^, maximal gradient 3 × 10^−4^, RMS gradient 1 × 10^−4^, maximal displacement 4 × 10^−3^, RMS displacement 2 × 10^−3^ a.u. Vibrational frequency calculations at the same level of theory confirmed that all structures are true minima (has no imaginary frequencies) and transition states feature exactly one imaginary frequency.

To assess the overall stability of molecules and compare isomers, we calculated the oxygen binding energy for the epoxy-functionalized coronene derivatives:

*BE_O_* = *U*_total_ − (*U*_D3h_ + *E*_O_),

where *U*_total_—internal energy of the system; *U*_D3h_—internal energy of the pristine GQD-D3h; *E*_O_—electronic energy of a single, isolated oxygen atom in triplet ground state. The internal energy was calculated as the sum of electronic energy and the zero-temperature vibrational energy:

*U* = *E*_el_ + *E*_ZPE_,

where *E*_el_—total energy from the electronic structure calculation; *E*_ZPE_—zero-temperature vibrational energy from the frequency calculation.

Excited state energies and optical absorption spectra were calculated for the optimized GQDs with oxygen groups using the simplified time-dependent density functional theory approach (sTD-DFT) [31,32] with a dispersion-corrected hybrid B3LYP-D3 density functional and 6-311G(d,p) basis set (std 2.0 program package). In combination with global hybrid density functionals, the sTD-DFT approach has shown good quantitative agreement with experiments for compounds with a valence-dominated response, such as conjugated π-systems [32,33]. The absorption spectra were simulated using the calculated transition energies in the range from 0 up to 8 eV (about 360 excited states) and corresponding oscillator strengths with a full width at half maximum of 0.15 eV.

## 4. Conclusions

It is demonstrated that ether bridge formation during the relaxation of epoxidized GQDs is governed by one of two structural conditions. The first condition involves epoxy attachment to a bond connecting two aromatic sextets. The second condition occurs when the epoxy group links a carbon atom of an aromatic sextet to a carbon atom in an adjacent benzene ring participating in localized π-bond formation. When these conditions are met, C–C bond cleavage prevents aromatic sextet disruption, thereby substantially enhancing the thermodynamic stability of the system.

Conversely, the attachment of an epoxy group to two carbon atoms of an aromatic sextet (one of which is shared between two benzene rings) leads to a significant decrease in structural stability. In this case, transformation into an ether bridge does not prevent the loss of the aromatic sextet but enables the migration of the remaining sextets. The combination of these factors explains the presence of an extremely low activation barrier for the transformation.

It is also demonstrated that the epoxy group and the ether bridge exert a comparable effect on the electronic and optical characteristics of GQDs. Furthermore, the ether bridge can generally be regarded as the final transformation product, whereas the epoxy group represents an intermediate species that may undergo significant changes upon contact with the environment. Thus, for the controlled tuning of the electronic and optical properties of GQDs via epoxidation, one should primarily consider metastable positions, as well as configurations lacking a local energy minimum. The identification of such positions is proposed using Clar’s rule, which predicts the propensity for C–C bond cleavage without additional quantum-chemical calculations.

## Figures and Tables

**Table 1:** Absolute and relative oxygen binding energy (to most favorable configuration—epoxy-α), energy gap, symmetry group of epoxidized GQD-D3h, energy barrier for epoxy → ether transformation and corresponding Gibbs free energy of activation.

Oxygen Position	Binding Energy (kcal/mol)	Relative Binding Energy (kcal/mol)	Energy Gap (eV)	Symmetry Group	Transformation Barrier (kcal/mol)	Gibbs Free Energy of Activation (kcal/mol)
epoxy-α	−65.7	—	2.93	C_S_	44.7	42.8
ether-β	−62.5	3.2	2.91	C_1_	—	—
epoxy-γ	−42.0	23.7	2.58	C_1_	0.2	0.2
epoxy-δ	−63.6	2.1	2.83	C_1_	49.0	47.2
epoxy-ε	−43.6	22.1	2.49	C_1_	0.4	−0.1
ether-ζ	−60.6	5.1	2.88	C_S_	—	—

**Table 2:** Oxygen binding energy, energy gap, and symmetry group for positions ether-γ and ether-ε in GQD-D3h.

Oxygen Position	Binding Energy (kcal/mol)	Energy Gap (eV)	Symmetry Group
ether-γ	−60.6	2.84	C_1_
ether-ε	−62.5	2.83	C_1_

**Table 3:** Energy gap and optical properties (excitation energies, wavelengths, oscillator strengths and transition components for first two singlet excited states) of epoxidized GQD-D3h at various positions.

Structure	Energy Gap (eV)	Excited Singlet	Excitation Energy (eV)	Wavelength (nm)	Oscillator Strength	Transition Components
epoxy-α	2.93	S_1_	2.49	498	0.17	H → L (91%)
		S_2_	2.50	496	0.00	H-1 → L (54%); H → L + 1 (46%)
ether-β	2.91	S_1_	2.45	506	0.11	H → L (67%)
		S_2_	2.48	500	0.05	H-1 → L (37%); H → L + 1 (36%)
ether-γ	2.84	S_1_	2.37	523	0.04	H-1 → L (38%); H → L + 1 (38%)
		S_2_	2.43	510	0.14	H → L (67%)
epoxy-δ	2.83	S_1_	2.39	519	0.02	H-1 → L (48%); H → L + 1 (42%)
		S_2_	2.42	512	0.18	H → L (80%)
ether-ε	2.83	S_1_	2.38	521	0.08	H → L (40%); H → L + 1 (28%)
		S_2_	2.41	514	0.11	H → L (50%); H-1 → L (22%)
ether-ζ	2.88	S_1_	2.46	504	0.23	H → L (93%)
		S_2_	2.48	500	0.00	H-1 → L (55%); H → L + 1 (45%)

## Data Availability

All data that supports the findings of this study is available in the published article and/or the Supplementary Materials of this article.

## Supplementary Materials

The following supporting information can be downloaded at https://www.mdpi.com/article/10.3390/molecules31132269/s1.

## Abbreviations

The following abbreviations are used in this manuscript:

GQD	Graphene Quantum Dot
PAH	Polycyclic Aromatic Hydrocarbon
FMO	Frontier Molecular Orbital
HOMO	Highest Occupied Molecular Orbital
LUMO	Lowest Unoccupied Molecular Orbital
DFT	Density Functional Theory
B3LYP	Becke and Lee–Yang–Parr Hybrid Density Functional
sTD-DFT	Simplified Time-Dependent DFT
RMS	Root Mean Square
TST	Transition State Theory
NEB	Nudged Elastic Band

## References

[1] Li X., Rui M., Song J., Shen Z., Zeng H. (2015). Carbon and Graphene Quantum Dots for Optoelectronic and Energy Devices: A Review. Adv. Funct. Mater..

[2] Kalluri A., Dharmadhikari B., Debnath D., Patra P., Kumar C.V. (2023). Advances in Structural Modifications and Properties of Graphene Quantum Dots for Biomedical Applications. ACS Omega.

[3] Zheng X.T., Ananthanarayanan A., Luo K.Q., Chen P. (2014). Glowing Graphene Quantum Dots and Carbon Dots: Properties, Syntheses, and Biological Applications. Small.

[4] Tian P., Tang L., Teng K.S., Lau S.P. (2018). Graphene Quantum Dots from Chemistry to Applications. Mater. Today Chem..

[5] Feng J., Dong H., Yu L., Dong L. (2017). The Optical and Electronic Properties of Graphene Quantum Dots with Oxygen-Containing Groups: A Density Functional Theory Study. J. Mater. Chem. C.

[6] Sheely A., Gifford B., Tretiak S., Bishop A. (2021). Tunable Optical Features of Graphene Quantum Dots from Edge Functionalization. J. Phys. Chem. C.

[7] Cui P., Xue Y. (2022). Tuning Nonradiative Recombination Loss by Selective Oxidation Patterns of Epoxy Groups Bound to Different Sites of Graphene Quantum Dots. Chem. Eng. J..

[8] Park M., Kim H.S., Yoon H., Kim J., Lee S., Yoo S., Jeon S. (2020). Controllable Singlet–Triplet Energy Splitting of Graphene Quantum Dots through Oxidation: From Phosphorescence to TADF. Adv. Mater..

[9] Chung S., Revia R.A., Zhang M. (2019). Graphene Quantum Dots and Their Applications in Bioimaging, Biosensing, and Therapy. Adv. Mater..

[10] Ershov I.V., Lavrentyev A.A., Romanov D.L., Holodova O.M. (2025). Tuning Optical Excitations of Graphene Quantum Dots through Selective Oxidation: Effect of Epoxy Groups. C.

[11] Yan J.-A., Xian L., Chou M.Y. (2009). Structural and Electronic Properties of Oxidized Graphene. Phys. Rev. Lett..

[12] Kim S., Zhou S., Hu Y., Acik M., Chabal Y.J., Berger C., de Heer W., Bongiorno A., Riedo E. (2012). Room-Temperature Metastability of Multilayer Graphene Oxide Films. Nat. Mater..

[13] Li Z., Zhang W., Luo Y., Yang J., Hou J.G. (2009). How Graphene Is Cut upon Oxidation?. J. Am. Chem. Soc..

[14] Romanov D., Lavrentyev A., Ershov I. (2025). Application of Clar’s Rule for Assessing the Effect of an Epoxy Group on the Stability and Energy Gap of Graphene Quantum Dots: A Coronene-Based DFT Study. Chemistry.

[15] Clar E. (1972). The Aromatic Sextet.

[16] Balaban A.T., Klein D.J. (2009). Claromatic Carbon Nanostructures. J. Phys. Chem. C.

[17] Solà M. (2013). Forty Years of Clar’s Aromatic π-Sextet Rule. Front. Chem..

[18] Rabeya R., Mahalingam S., Manap A., Satgunam M., Akhtaruzzaman M., Chia C.H. (2022). Structural Defects in Graphene Quantum Dots: A Review. Int. J. Quantum Chem..

[19] Kastler M., Schmidt J., Pisula W., Sebastiani D., Müllen K. (2006). From Armchair to Zigzag Peripheries in Nanographenes. J. Am. Chem. Soc..

[20] Rieger R., Müllen K. (2010). Forever young: Polycyclic aromatic hydrocarbons as model cases for structural and optical studies. J. Phys. Org. Chem..

[21] Ershov I.V., Lavrentyev A.A., Bazhin I.V., Holodova O.M., Prutsakova N.V., Zhdanova T.P., Romanov D.L. (2023). Modelling the Structure and Optical Properties of Reduced Graphene Oxide Produced by Laser Ablation: Insights from XPS and Time-Dependent DFT. Crystals.

[22] Yoon H., Park M., Kim J., Novak T.G., Lee S., Jeon S. (2021). Toward highly efficient luminescence in graphene quantum dots for optoelectronic applications. Chem. Phys. Rev..

[23] Makkar P., Ghosh N.N. (2021). A Review on the Use of DFT for the Prediction of the Properties of Nanomaterials. RSC Adv..

[24] Pitman S.J., Evans A.K., Ireland R.T., Lempriere F., McKemmish L.K. (2023). Benchmarking Basis Sets for Density Functional Theory Thermochemistry Calculations: Why Unpolarized Basis Sets and the Polarized 6-311G Family Should Be Avoided. J. Phys. Chem. A.

[25] Becke A.D. (1993). Density-Functional Thermochemistry. III. The Role of Exact Exchange. J. Chem. Phys..

[26] Ásgeirsson V., Orri Birgisson B., Björnsson R., Becker U., Neese F., Riplinger C., Jonsson H. (2021). Nudged Elastic Band Method for Molecular Reactions Using Energy-Weighted Springs Combined with Eigenvector Following. J. Chem. Theory Comput..

[27] Bykov D., Petrenko T., Izsak R., Kossmann S., Becker U., Valeev E., Neese F. (2015). Efficient implementation of the analytic second derivatives of Hartree-Fock and hybrid DFT energies: A detailed analysis of different approximations. Molec. Phys..

[28] Eyring H. (1935). The Activated Complex in Chemical Reactions. J. Chem. Phys..

[29] Grimme S., Antony J., Ehrlich S., Krieg H. (2010). A consistent and accurate ab initio parametrization of density functional dispersion correction (DFT-D) for the 94 elements H-Pu. J. Chem. Phys..

[30] Neese F. (2022). Software update: The ORCA program system, version 5.0. WIRES Comput. Mol. Sci..

[31] Grimme S. (2013). A simplified Tamm-Dancoff density functional approach for the electronic excitation spectra of very large molecules. J. Chem. Phys..

[32] Bannwarth C., Grimme S. (2014). A simplified time-dependent density functional theory approach for electronic ultraviolet and circular dichroism spectra of very large molecules. Comput. Theor. Chem..

[33] Wergifosse M., Seibert J., Grimme S. (2020). Simplified time-dependent density functional theory (sTD-DFT) for molecular optical rotation. J. Chem. Phys..

