# Supplementary material for: From Intermediate Epoxy Group to Stable Ether Bridge: Insights from DFT Study on Graphene Quantum Dots
Source: Molecules. 2026 Jun 29;31(13):2269. doi: 10.3390/molecules31132269 (PMC13362549; doi:10.3390/molecules31132269)
Supplement: Supplementary file 1 [file molecules-31-02269-s001.zip › molecules-4372891-supplementary.pdf]

SUPPORTING INFORMATION

for

From Intermediate Epoxy Group to Stable Ether Bridge: Insights from a DFT Study on Graphene Quantum Dots

Dmitry Romanov, Anatoly Lavrentyev, Igor Ershov

TABLE OF CONTENTS

S1. Transformation of an Epoxy Group Into an Ether Bridge....................2

S2. Optical Properties....................3

S3. Geometry Convergence Plot....................4

S4. Density of States....................5

S5. Frontier Molecular Orbital Distribution....................6

S6. Benzo[a]pyrene as a Model of GQD....................7

S7. Binding Energy....................8

S1. Transformation of an Epoxy Group Into an Ether Bridge

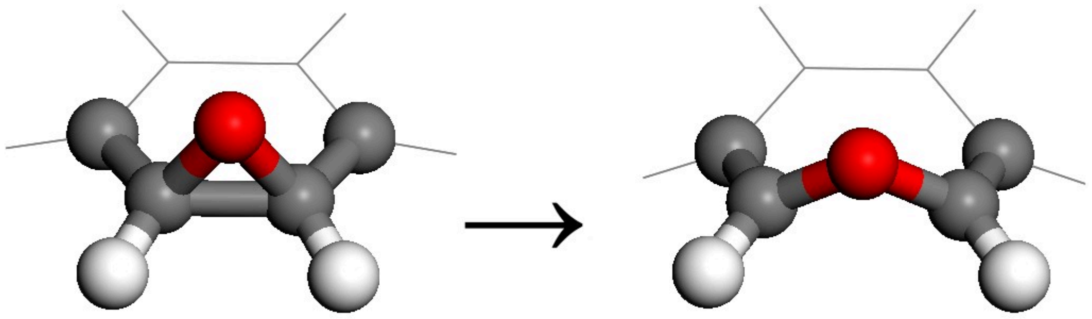

Figure S1. Scheme of transformation of an epoxy group into an ether bridge

S2. Optical Properties

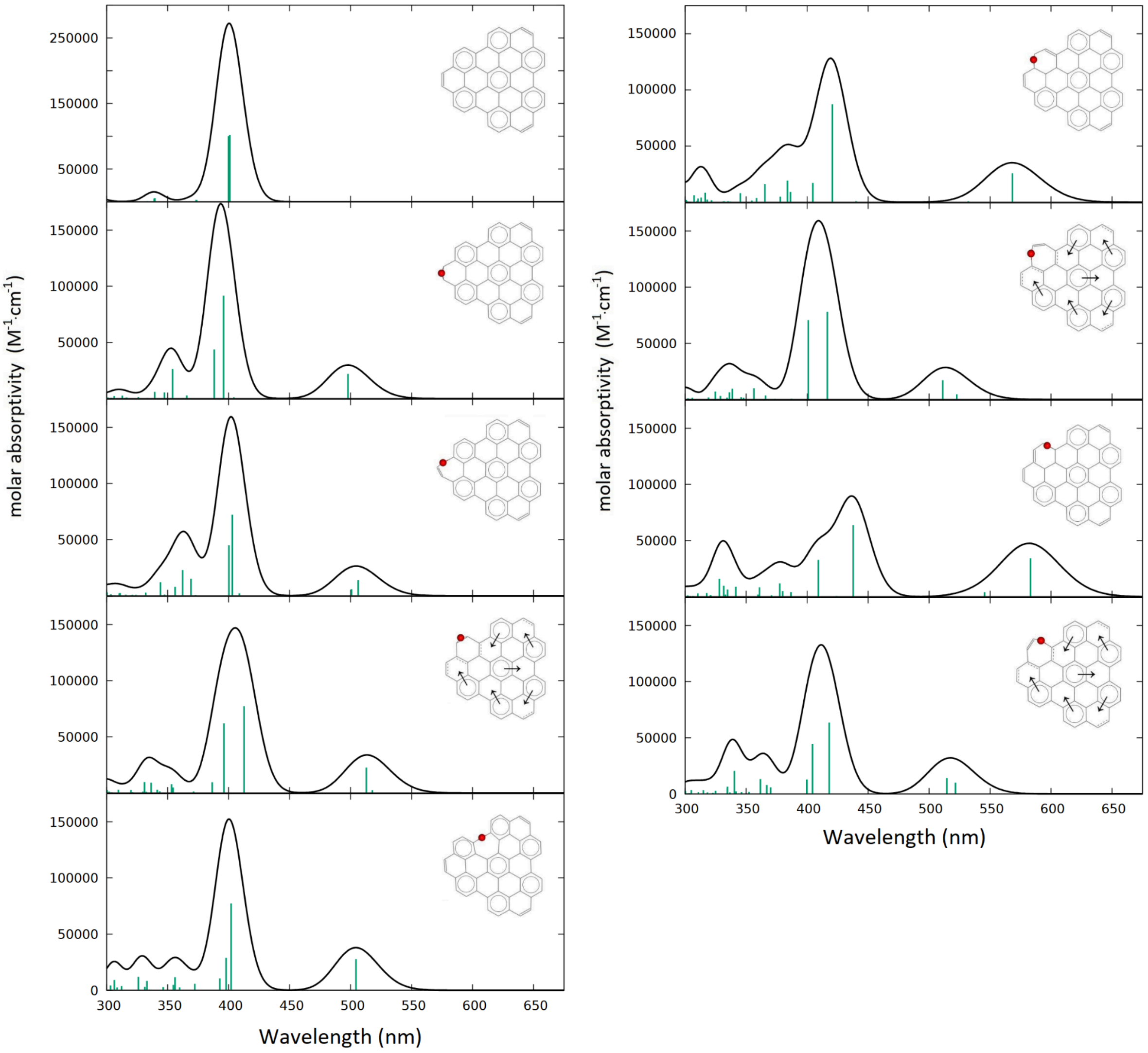

Figure S2. Optical absorption spectra of pristine and epoxidized GQD D3h structures at various functionalization positions.

S3. Geometry Convergence Plot

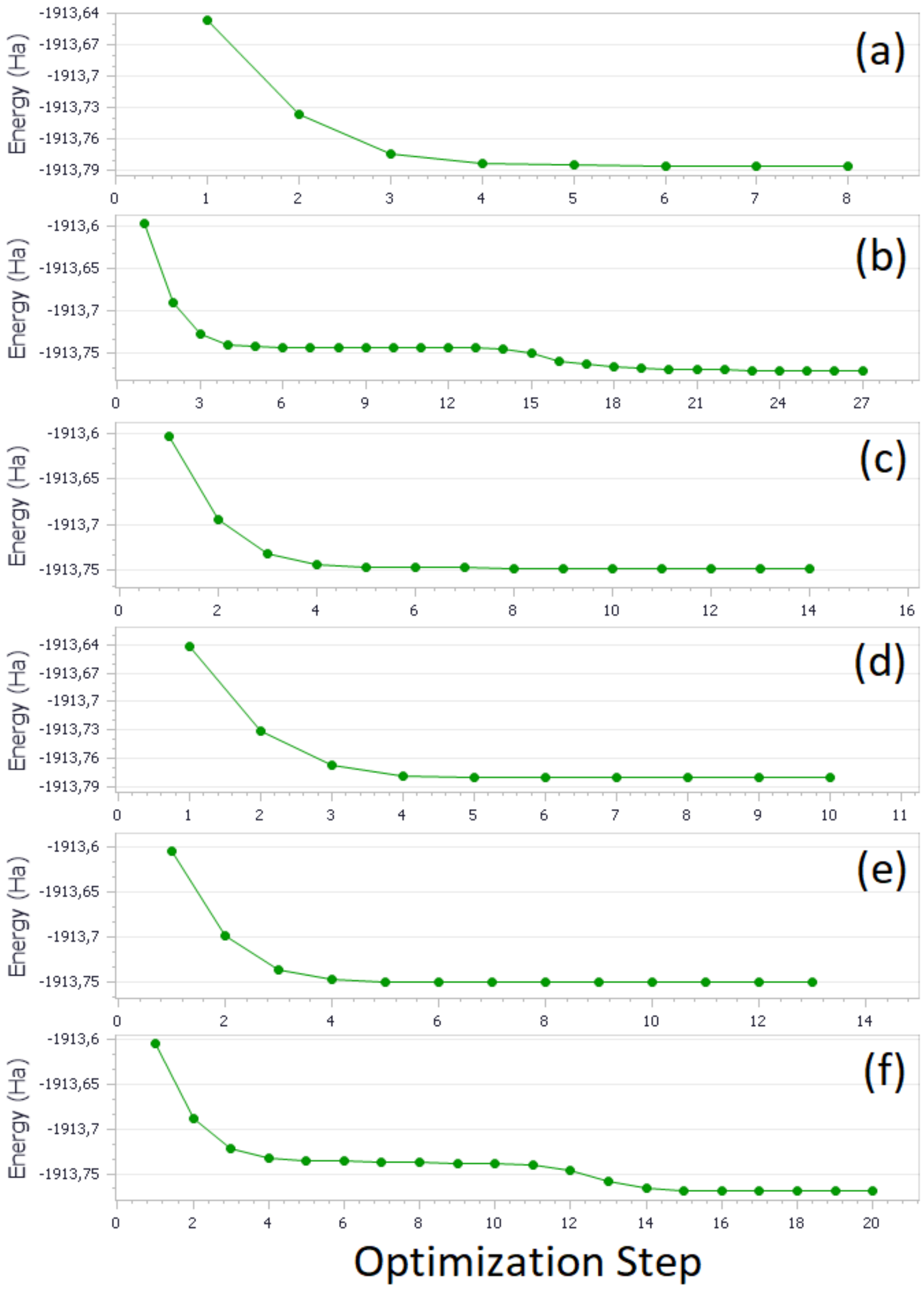

Figure S3. Geometry convergence plot (Total energy vs. optimisation step number) for the epoxidized D3h structure: (a) — epoxy-α, (b) — ether-β, (c) — epoxy-γ, (d) — epoxy-δ, (e) — epoxy-ε, (f) — ether-ζ.

S4. Density of States

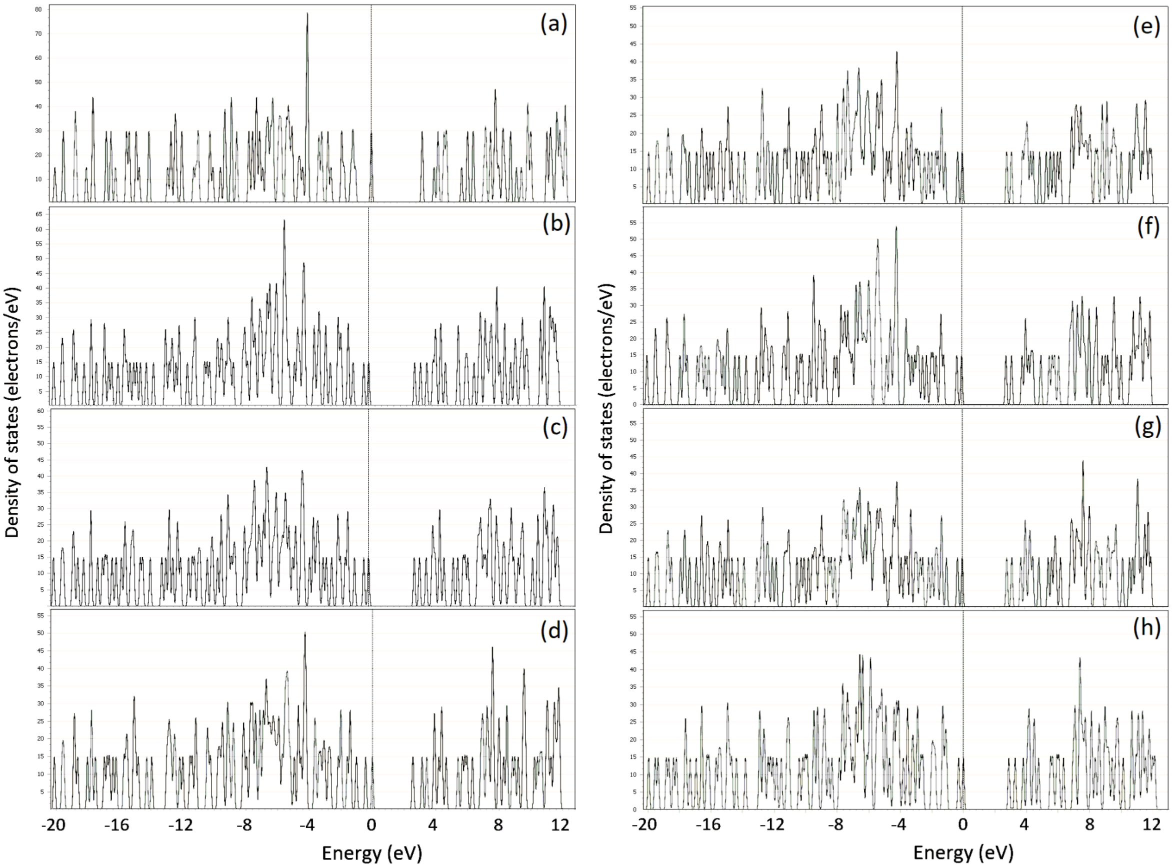

Figure S4. Total density of electronic states for pristine and epoxidized GQD-D3h at different positions: (a) — pristine structure, (b) — epoxy-α, (c) — ether-β, (d) — epoxy-γ, (e) — ether-γ, (f) — epoxy-δ, (g) — ether-ε, (h) — ether-ζ.

S5. Frontier Molecular Orbital Distribution

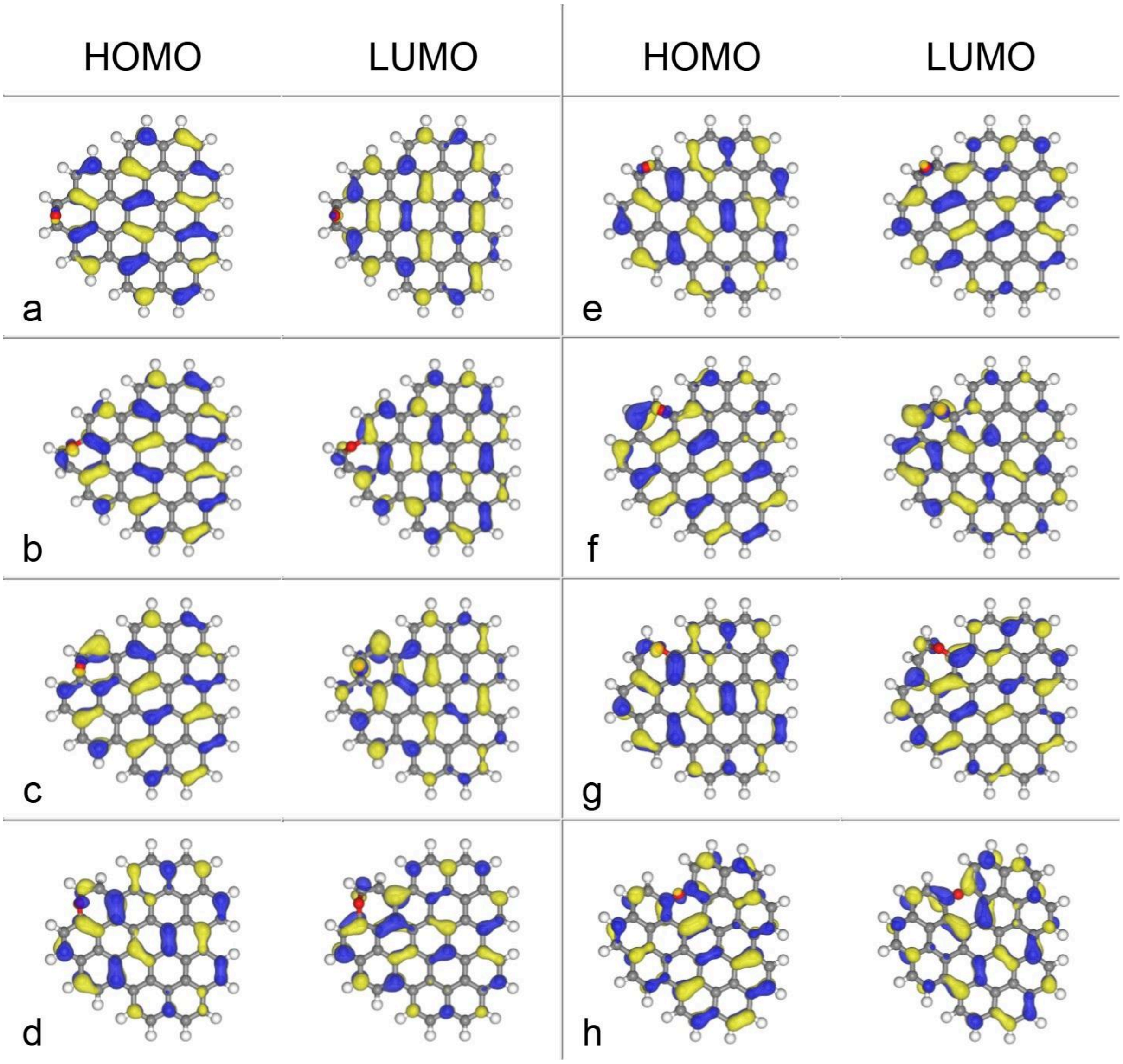

Figure S5. Frontier molecular orbital distribution for epoxidized GQD-D3h at different positions: (a) — epoxy-α, (b) — ether-β, (c) — epoxy-γ, (d) — ether-γ, (e) — epoxy-δ, (f) — epoxy-ε, (g) — ether-ε, (h) — ether-ζ.

S6. Benzo[a]pyrene as a Model of GQD

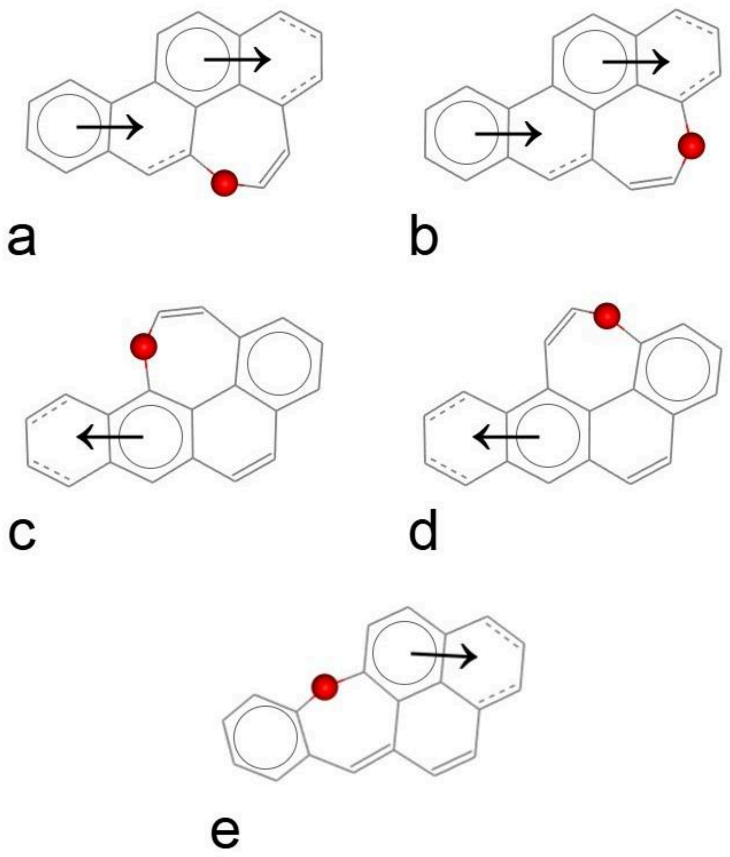

Figure S6. The epoxy group attachment sites on benzo[a]pyrene that yield ether bridges upon relaxation.

S7. Binding Energy

Table S1. Binding energy of pristine GQD-D3h and its epoxidized isomers.

Structure	Binding Energy (kcal/mol)
Pristine D_{3h}	- 87132.8
epoxy-α	- 89857.9
ether-β	- 89853.3
epoxy-γ	- 89832.9
ether-γ	- 89851.8
epoxy-δ	- 89856.5
epoxy-ε	- 89834.9
ether-ε	- 89853.2
ether-ζ	- 89851.1
